# Long-Term Care Managers’ Approaches to Quality Improvement Work in Service Planning and Provision: A Qualitative Interview Study

**DOI:** 10.1177/23333936251336093

**Published:** 2025-04-28

**Authors:** Randi Olsson Haave, Marianne Sundlisæter Skinner, Line Melby

**Affiliations:** 1 Centre for Care Research, NTNU – Norwegian University of Science and Technology, Gjøvik, Norway; 2 Department of Health Research, SINTEF Digital, Trondheim, Norway

**Keywords:** quality of health care, nursing homes, home care services, management, qualitative research, Norway, Kvalitet i helsetjenesten, sykehjem, hjemmesykepleie, ledelse, kvalitativ forskning, Norge

## Abstract

Long-term care managers are essential in creating high-quality services. Previous research has a limited focus on managers’ practices and approaches to quality improvement work. This study aims to explore how Norwegian long-term care managers approach systematic and ad hoc quality improvement work in the context of service planning and provision. In-depth interviews were conducted with twelve long-term care managers from three municipalities and analysed using inductive thematic analysis. Three themes were identified: *delegating responsibility to ensure care quality*, *the creation of comparable services is a goal* and *using quality indicators in quality improvement work*. The results indicate that using key nurses’ expertise and resources were crucial approaches in the long-term care managers’ quality improvement work. Quality indicators, delegation of responsibility to other care staff and collaboration within and across municipalities were used to varying degrees. Increased collaboration with other managers, involvement of care staff and awareness and use of quality indicators are approaches that may strengthen long-term care managers’ quality improvement work.

## Abstrakt

Ledere i kommunale helse- og omsorgstjenester har en nøkkelposisjon i arbeidet for å skape tjenester av god kvalitet. Tidligere forskning har i begrenset grad satt søkelys på ledernes praksiser og tilnærminger til kvalitetsforbedringsarbeid. Denne studien utforsker hvilke systematiske og ad hoc strategier ledere i kommunale helse- og omsorgstjenester benytter i kvalitetsforbedringsarbeid, under planlegging og utøvelse av tjenestene. Individuelle intervjuer ble gjennomført med tolv kommunale helse- og omsorgstjenesteledere i tre kommuner i Norge, og analysert med induktiv tematisk analyse. Tre temaer ble identifisert: *delegering av ansvar for å sikre kvaliteten på tjenestene*, *sammenlignbare tjenester er et mål* og *bruk av kvalitetsindikatorer i kvalitetsforbedringsarbeid*. Resultatene indikerer at fagutviklingssykepleieres ekspertise og ressurser var avgjørende for lederne i deres kvalitetsforbedringsarbeid. Kvalitetsindikatorer, delegering av ansvar til tjenestens øvrige ansatte og samarbeid internt og på tvers av kommuner benyttet lederne derimot i varierende grad. Økt samarbeid med andre ledere innenfor tjenestene, involvering av tjenestenes ansatte samt kunnskap og bruk av kvalitetsindikatorer er strategier som kan styrke kommunale helse- og omsorgstjenestelederes kvalitetsforbedringsarbeid.

## Introduction

High-quality healthcare services are a primary goal for healthcare services internationally (OECD/WHO/World Bank Group, 2018; World Health Organization [WHO], 2025). Healthcare quality, however, is a complex phenomenon on which care recipients, next-of-kin, care staff, managers, policymakers and other stakeholders have different perspectives. Various definitions of quality in healthcare have been put forward, many of which involve aspects of recipients’ requirements, expectations and care experiences (Donabedian & Bashsur, 2003; Institute of Medicine, 2001). Internationally, the definition introduced by the Institute of Medicine (IOM) is frequently referred to, where high-quality healthcare refers to effective, safe and person-centred treatment provided by an integrated, equitable, efficient and timely healthcare system (Institute of Medicine, 2001; WHO, 2025).

Long-term care managers at different service levels are responsible for ensuring quality improvement work in their units, ranging from administrative obligations related to finances and staff to overall responsibility for the quality of recipients’ treatment and care. This article applies *quality improvement work* as a broad term, which includes systematic and unstructured or ad hoc quality improvement work carried out in the services’ day-to-day work.

Research on the role of long-term care managers in quality improvement work has adopted different perspectives. Some researchers have found that managers view ‘quality’ as a complex concept that encompasses more than measurable or concrete factors. They emphasise, for example, the importance of dignity, empowerment of the care recipients and the care staff’s tacit knowledge (Aase et al., 2021; Farr & Cressey, 2015; Yamamoto-Mitani et al., 2018). This complexity is also evident in managers’ challenges with quality improvement work, which involves care staff competence, documentation systems and finances – factors that impact service quality (Johannessen et al., 2020; Ree et al., 2019; Soreide et al., 2019). Other studies have focused on different models and interventions to enhance managers’ competence and role in quality improvement work (Havig et al., 2011; Johannessen et al., 2021). In addition, the manager’s role and importance in providing person-centred care (Backman et al., 2021) and how different leadership and management styles can influence service quality have been highlighted (Dawes & Topp, 2021; Nieuwboer et al., 2019). Despite recognising managers’ importance in quality improvement work, research on how long-term care managers contribute to providing high-quality services is limited (Asante et al., 2021; Orellana et al., 2017; Siegel & Young, 2021).

As a result of an increased focus on healthcare service quality, various quality indicators or measurements have been developed for use in long-term care services. Standardised quality indicators are used to compare healthcare systems, manage healthcare services and facilitate improvement work (OECD, 2019; Schneider et al., 2021). An expanding population of complex user groups with advanced healthcare needs in long-term care services has strengthened the focus on service quality and increased the use of quality indicators on these levels (Glette et al., 2018). This shift has led to an increased focus on the importance and position of quality indicators in quality improvement work at the local level (Schneberk et al., 2022; Sourial et al., 2022; WHO & UN, 2022). There have been calls for more research highlighting how long-term care managers contribute to and facilitate service quality improvement work (Orellana et al., 2017; Siegel et al., 2018; Wong et al., 2013). This research aimed to address this gap by *exploring how Norwegian long-term care managers approach systematic and ad hoc quality improvement work in the context of service planning and provision.*

## Methods

### Design

The study employed a descriptive, explorative, qualitative approach (Sandelowski, 2010) suited for understanding experiences of phenomena such as long-term managers’ approach to quality improvement work. This research was inspired by a social constructivist paradigm, as interactions between people in their context shape their experiences (Berger & Luckmann, 1971). The results from this study were reported based on standards for reporting qualitative research (Tong et al., 2007).

### Study Context

In Norway, the healthcare system is mainly publicly funded by taxes and co-payments. The national government has delegated responsibility for organising and providing primary healthcare and long-term care, such as nursing homes and home care services, to the municipalities. The organisation of long-term care services varies between municipalities, as they are free to structure the services based on local conditions, needs and opportunities (Saunes et al., 2020; Tikkanen et al., 2020). However, nursing homes and home care services are normally organised into ‘units’, where nursing home units are organised according to length of stay, diagnoses or particular care needs (e.g., rehabilitation and dementia) and home care services are structured into units according to geographical area. Nursing home and home care staff have different backgrounds and levels of health education, and nonphysician staff groups are commonly characterised by part-time positions (The Norwegian Directorate of Health, 2021).

Awareness of the quality of healthcare services has increased in Norway since the 2000s due to national and international attention to healthcare quality and the decentralisation of healthcare services from specialist healthcare to municipal healthcare services (Institute of Medicine, 2001; The Norwegian Directorate of Health, 2018). This decentralisation means that municipal long-term care services have been given responsibility for providing and coordinating care for care recipients with more complex needs than before (St. Meld. 47, 2008–2009). The care quality objectives of the Norwegian health and care service are anchored in national policy documents and the IOM’s definition of quality (Institute of Medicine, 2001; The Norwegian Directorate of Health, 2018). They state that the municipalities are required to provide safe, equitable, effective and person-centred care provided by a professionally sound service that focuses on quality improvement. Health authorities have implemented several measures to improve care quality, and outcomes are measured in several ways.

Quality indicators are indirect measures of the quality of healthcare services, and numerous measures and campaigns have been implemented to strengthen service quality (The Norwegian Directorate of Health, 2022). To improve service quality, long-term care services are also encouraged to use measurement tools included in a national patient safety framework, such as malnutrition and pressure ulcer screenings (The Norwegian Directorate of Health, n.d.). The indicators used in Norwegian long-term care services are reported in two nationwide databases: the Norwegian Registry for Primary Health Care and Municipality-State Reporting (Helsedata, 2024; Statistics Norway, 2023a). Municipalities must report figures to the registries annually, and many municipalities have integrated the Norwegian Registry for Primary Health Care into their electronic patient record systems. The quality indicator results are used as an information source for management decisions, comparisons and quality improvement work on the municipal and national levels (The Norwegian Directorate of Health, 2022). However, the health authorities warn that results must be applied and interpreted with caution, as there are different reporting practices and under-reporting in the municipalities (The Norwegian Directorate of Health, 2022). Contrary to other countries (Eckhardt et al., 2019), in Norway, long-term care services are not ranked or rewarded if they exceed expected quality targets.

The regulation ‘Management and Quality Improvement in the Health and Care Services’ (The Norwegian Directorate of Health, 2017) also stipulates that managers at all levels are responsible for delivering professionally sound services of high quality. Therefore, managers must continuously focus on various aspects of quality improvement work, such as development of procedures, application of indicators, routines for care staff training and deviation management, to achieve national quality ambitions. In addition to fulfilling their own responsibilities, the managers must facilitate the care staff’s fulfilment and integration of quality improvement work in their unit’s care practices (The Norwegian Directorate of Health, 2017). However, reports have shown that despite national guidelines and objectives for quality, quality improvement work in long-term care services is less systematic and draws on fewer quality indicators than in specialist healthcare (Tikkanen et al., 2020).

### Setting and Study Participants

Three municipalities were included in this study and recruited through digital presentations by the first author at online conferences. Following these presentations, representatives from three municipalities contacted the first author and registered their interest in participating. Thereafter, one municipality-level healthcare service manager from each registered municipality was sent an e-mail invitation to participate and asked to forward the invitation to the other healthcare service managers (at different levels) in the municipality. A total of 12 managers registered their interest in participating and were included in the study. The characteristics of the participants are presented in Table 1. Out of the 12 participants, there were, respectively, three, four and five participants from each of the municipalities. The participants operated at three different management levels: they were either managers of one home care or nursing home unit (level 1), several home care or nursing home units (level 2) or managers of a large section of the long-term care services in the municipality (level 3) (Table 1).

**Table 1.** Participant Characteristics.

	Participant number (P) and educational background^1^	Experience as manager (years)^2^	Service^3^	Level^4^	Number of FTEs^5^
**Municipality 1**	P 1, RN	8	NH	1	29
	P 2, RN	10	NH	1	19
	P 3, RN	25	NH	1	45
	P 4, RN	12	ML (NH)	3	220
**Municipality 2**	P 5, ME	26	ML (NH, HC)	3	570
	P 6, RN	10	NH	2	87
	P 7, RN	No previous experience	NH	2	25
	P 8, RN	6	NH	2	174
	P 9, RN	35	NH, HC	2	96
**Municipality 3**	P 10, LDN	12	NH	2	33
	P 11, RN	10	HC	1	21
	P 12, LDN	20	ML (HC)	3	109

*Note*. ^1^RN = registered nurse; ME = management education; LDN = learning disability nurse.

2 Years as manager.

3 HC = home care unit(s); NH = nursing home unit(s); ML = municipality level.

4 Levels of responsibility, level 1: responsibility for a nursing home or home care unit; level 2: responsibility for several nursing homes or home care units; level 3: overall responsibility for a larger portion of the long-term care services in the municipality.

5 Responsibility for the number of full-time equivalents (FTEs).

Most of the participants, like many long-term care managers in Norway, had backgrounds as registered nurses or other healthcare professionals, such as learning disability nurses or social workers (Glette et al., 2018). Some of them had additional education in administration and management. None of the managers were responsible for providing direct care to the recipients. Still, those holding responsibility for one or more home care or nursing home units (level 1 or 2) had offices located in the units and were, therefore, closer to the unit’s day-to-day care. Managers’ responsibilities depended on the management level, but all were responsible for budgets and human resources. All the managers were responsible for quality improvement work within the services, irrespective of managerial level, and were bound by the regulation ‘Management and Quality Improvement in the Health and Care Services’ (Ministry of Health and Care Services, 2017; Saunes et al., 2020). All participants except one were women.

The three municipalities included in the study comprised urban and rural areas with different geographical characteristics, such as size and population. The municipalities’ populations ranged from approximately 14,000 to 100,000 (Statistics Norway, 2023b). All three municipalities reported on quality indicators to the national registries and used measurement tools (such as screenings for pressure ulcers, malnutrition or fall risk) from the national patient safety framework (The Norwegian Directorate of Health, 2022, n.d.).

### Data Collection

The interviews took the form of in-depth individual interviews. An interview guide was prepared based on previous studies of quality improvement work, the use of quality indicators and the quality indicators developed for Norwegian long-term care services. This guide included open-ended questions about the quality improvement work performed in the organisation, perspectives regarding the concepts of quality and quality indicators, routines for receiving new care recipients, social activities in the unit, nutrition screening and various types of user involvement (Supplementary file 1). In addition, the participants were asked to describe their current position and managerial experience. The interviews were conducted from January to May 2022 and lasted between 48 and 79 min each (average 60 min). The digital platform Teams^®^ was used for the interviews, but a voice recorder was used instead of the video and audio software integrated into the digital platform. All interviews were conducted by the first author and transcribed verbatim by a research assistant.

Twelve individual interviews were arranged and carried out. During and after the data collection, we reflected on the material’s information richness, drawing on Malterud et al.’s (2016) and Braun and Clarke’s (2022a) descriptions of information power. The participants’ extensive descriptions and examples of approaches to strengthen service quality, their active communication with the first author during the interviews and the choice of an in-depth analysis strategy made us consider the data material as rich, in line with Malterud et al.’s (2016) conceptualisation of information power. After conducting the twelve interviews, we, therefore, concluded that the data material had sufficient richness and proceeded with the analysis.

### Data Analysis

A thematic analysis was conducted to analyse the study participants’ approaches to quality improvement work in the context of service planning and provision (Braun & Clarke, 2022b). Initially, the transcripts and the recordings were read and listened to repeatedly by the first author to achieve a deep understanding of the material. An inductive and text-driven approach was used. After these initial readings, the entire material was thoroughly reread sentence by sentence, and text sections were coded. Semantic coding was used in this part of the analysis to identify the participants’ approaches to quality improvement work (Braun & Clarke, 2022b). The first author then grouped the codes thematically under preliminary themes identified during the reading and coding process. This part of the analysis featured transitions back and forth among the raw data, codes and initial themes to ensure coherence among these elements. After the new readings, some codes were recoded and grouped into three themes. The themes (and associated text) were reviewed in collaboration with the coauthors to determine the final themes. Latent analysis within each theme was used to gain further insights into the data, and the results were discussed and interpreted by all the authors to ensure trustworthiness. Table 2 presents the codes, subthemes and themes. NVivo® version 12 was used to support the analysis and coding process.

**Table 2.** Overview of the Codes, Sub-Themes and Themes.

Codes	Subthemes	Themes
Key nurses are delegated a variety of responsibilities and tasks Key nurses have an essential role in the quality improvement work	Key nurses are delegated responsibility for ensuring overall care quality	Delegating responsibility to ensure care quality
The right competence in care staff increases care quality Broad competence is needed to meet the complexity and diversity in care needs	Care staff’s competence is used to ensure individualised care	
Comparison of care quality across units is desirable but challenging Consistent and standardised practices with procedures increase care quality	Standardisation to improve service quality	The creation of comparable services is a goal
Support and knowledge exchange between care staff Committees, meetings and forums are valuable resources	Collaboration across and within municipalities	
Care staff competence development through meetings and seminars Different reporting routines and practices Discretionary assessments produce varying results	Reporting of quality indicators varies	Using quality indicators in quality improvement work
Quality indicators as an information source describing recipients’ care needs Quality indicators are complex and have limitations Quality indicators cannot capture the diversity of recipients’ care trajectories	Using quality indicators has benefits and limitations	

The study participants used the term ‘care staff’ to refer to registered nurses, assistant nurses and unskilled staff. In all the units, one or more registered nurses were responsible for quality improvement work and many other care-related and administrative tasks. The participants used titles such as ‘specialist nurse’, ‘nurse one’ or ‘team leader’ for these nurses, which are all job titles that imply that the nurses held a leading position in the care staff group. Their responsibilities typically include having overall responsibility for the quality of care, facilitating the professional development of other care staff, and promoting evidence-based practices. Personnel and financial responsibilities are not part of these nurses’ job descriptions. In reporting the results, the term ‘key nurse’ is used as a collective term for these positions. To support transparency and trustworthiness, the exact title of these nurses’ positions is included in the quotations from study participants.

### Rigour and Trustworthiness

Ongoing member checks or participant feedback during the interviews was implemented to strengthen the study’s credibility (Braun & Clarke, 2022b; Lincoln & Guba, 1985). Additional questions and tentative summaries of the participant’s descriptions were shared with them to ensure that their accounts were interpreted correctly. To enhance transferability, thick descriptions were used, inspired by an authentic narrative style (Younas et al., 2023). In the reporting of the findings, quotes are used to describe and illustrate how the participants approached quality improvement work to strengthen the quality of the services (Polit & Beck, 2021). The first author conducted all the interviews and used a guide with predefined topics to ensure consistency in the data collection and the study’s dependability (Lincoln & Guba, 1985). This author was a PhD student and a registered nurse. All three authors discussed the results throughout the analysis to improve confirmability and maintain participants’ perspectives in the interpreted findings.

### Ethical Considerations

The study was conducted in line with the principles of the Declaration of Helsinki and was approved by the Norwegian Agency for Shared Services in Education and Research (Sikt) (Ref. No. 672888). All study participants were sent an information letter containing details about the study’s purpose, background and protection of anonymity, as well as their option to withdraw. The information letter also included a consent form, which the participants completed. Information regarding the study’s purpose was repeated at the start of each interview, and the participants were encouraged to ask questions.

## Results

In the interviews, the participants underlined the importance of quality improvement work and explained that high-quality care and treatment was one of their main aims as long-term care managers. However, although the managers emphasised the importance of care service quality and quality improvement in their work, they described that they had insufficient time to do this work and, therefore, delegated it to others. The analysis identified three themes representing long-term care managers’ approaches to systematic and ad hoc quality improvement work in the context of service planning and provision: *delegation of responsibility is decisive to ensure care quality*, *the creation of comparable services is a goal* and *reporting and applying quality indicators is desired but demanding.* Table 2 shows the three themes and associated subthemes.

### Delegating Responsibility to Ensure Care Quality

The study participants used the key nurses’ and care staff’s resources and expertise in various ways in the units’ quality improvement work. Overall quality improvement tasks, such as implementing new routines and guidelines, were delegated to the key nurses as they, due to experience and training, were in a position to take on such responsibilities. Other care staff were assigned quality improvement tasks directly related to the recipient’s care, aligning with their competencies and other responsibilities.

#### Key Nurses Are Delegated Responsibilities for Ensuring Overall Care Quality

All study participants had key nurses in their staff that they delegated responsibility for ensuring the overall quality of care in the setting. This delegation of responsibility occurred irrespective of variations in number of these nurses, their roles and responsibilities as well as the allocated resources to carry out quality improvement work. Some participants recognised that key nurses had to carry out this work without allocated time. One participant described challenges related to key nurse’s working conditions as follows:

. . . [the nurse one position] was established because. . .all units were supposed to have this, but no resources were set aside for it. Then, it almost goes without saying that there isn’t much they [the nurse one] can do. . .. (Participant 9)

The participants described the key nurses in their units as ‘bridge builders’, ‘fire extinguishers’, role models and ‘cornerstones’. Due to the key nurses’ quality improvement work and competence, they were seen as crucial for ensuring and strengthening the quality of the unit’s everyday services. However, the participants also highlighted the importance of the key nurses in patient care and overall responsibilities in administering the unit’s services. One participant described the key nurses’ extensive areas of responsibility this way:

They [the specialist nurses] cover for me when I am not here. . .. Then, they carry the phone that the allocation office rings us on, and the person who has the phone is responsible for patient logistics. . .. They are out in the unit to a greater extent than I have time for. . . They participate in [interprofessional and nursing] reports, evaluation meetings, group meetings and so on, and they can identify issues in that way, such as training needs [among care staff]. They get suggestions for topics for professional meetings from the care staff, hire lecturers and organise the meetings. (Participant 3)

Several participants stated that key nurses’ responsibilities could be extensive and that prioritising quality improvement work could be challenging. At the same time, they recognised that these extensive work tasks gave the key nurses knowledge about the unit’s services, which they, in turn, could use to evaluate and initiate further quality initiatives.

Collaboration with other service providers within and across service levels was another area where participants expressed that they delegated responsibility to the key nurses. The allocation office, the municipality’s case and system managers, and other healthcare units were highlighted as essential collaboration partners to ensure quality and continuity throughout the recipients’ care trajectory. Several participants emphasised that, in parallel with the information exchanges with these partners, the key nurses also passed on information to relevant care staff in the units. This distribution of information was essential to provide high-quality care and treatment for the recipients. Summarised, participants were able to delegate responsibility to key nurses in multiple areas due to key nurses competence in varied areas such as direct quality improvement work, administration and coordination of the unit.

#### Care Staff’s Competence Is Used to Ensure Individualised Care

In contrast to the contributions of the key nurses, participants described to a lesser extent that they assigned quality improvement work to other care staff. However, the participants described the care staff’s knowledge and competence as crucial for the quality of the services.

They highlighted that a sufficient number of competent care staff in the unit was crucial for direct care and treatment, as care recipients had more complex diseases and were more fragile. One participant reflected on the care staff’s competence this way,

. . .We must have the right expertise, and we must have sufficient staffing based on the complexity and size of the patient group. (Participant 5)

Various diagnoses related to dementia and mental disorders were identified as conditions that demanded specific care staff expertise to care for the individual recipient. The complexity of these diagnoses required the care staff to adapt the treatment and care based on the individual care recipient’s needs. The participants highlighted that this individualisation, which was an example of an ad hoc quality improvement task, could strengthen service quality. The care staff’s high level of competence also entailed that many care recipients with multiple diagnoses and compound needs did not need to be transferred to a higher level of care in case their conditions worsened but could be treated and cared for at the unit. Resource groups of care staff or resource persons linked to dementia, palliative care and rehabilitation were also mentioned by some participants as resources.

### The Creation of Comparable Services Is a Goal

The study participants highlighted the importance of creating standardised ‘best’ practices to achieve comparable services and harmonisation across units or municipalities as a central part of the quality improvement work. Participants used two approaches to achieve comparable services. The first approach involved standardisation of practice and comparisons of the care provision and treatment across units and municipalities. The overall purpose of the standardisation and comparisons was the goal of providing equally high-quality services irrespective of geographical area. The second approach involved facilitating collaboration among care staff across and within care settings to enable sharing of knowledge and resources to enhance care quality. Each approach is discussed in more detail below.

#### Standardisation to Improve Service Quality

Harmonisation and standardisation of services across the municipality were a focus area for the participants. They described nursing coverage, financing, sick leave among care staff, care needs and the number of recipients as variables that were compared within or across units in the municipality. The comparisons of such variables were a vital part of standardising the services and creating equitable and high-quality healthcare throughout the municipality.

However, participants noted that they lacked information on several variables that represented the units’ practice; thus, they questioned the value of these comparisons. One participant shared the following reflection:

When we have higher competence, does it give us less absence from work, better patient flow or fewer readmissions? There are many variables and factors I feel we don’t know enough about when we compare ourselves with ourselves and that we need to work on more in the future. (Participant 5)

Aligning procedures for treatment was another example of creating consistent and standardised practices across units. Several participants argued that coordination and development of routines and procedures could contribute to more comparable services. One municipality had developed an overarching procedure for quality targets in healthcare services to create consistent, high-quality practices across units. Participants from this municipality explained that this procedure was not frequently discussed but that its corresponding focus areas guided the units’ quality improvement work.

#### Collaboration Across and Within Municipalities

The participants argued that collaboration, knowledge exchange and teaching activities across and within municipalities and organisations contributed to higher service quality. Co-locating units and establishing various meeting points and committees were approaches to enhance cooperation and knowledge transfer. Quality committees or groups and nurse meetings were forums where routines and procedures were managed, developed or reviewed. Nevertheless, due to a lack of time and resources, the work agreed upon in these forums was not always implemented, or meetings were cancelled. One participant explained the challenges faced by the quality committee as follows:

. . . if there are more general routines that we think apply to everyone, then they should be reviewed in the quality committee so that everyone in the services can use them. But we haven’t quite got this quality committee in place. Maybe it sometimes is, unfortunately, given lower priority because it is very busy in the units. I think perhaps we’ve had two quality meetings [in 16 months], and we are supposed to have 6-8 meetings a year. (Participant 11)

Some participants described these various meetings as important interdisciplinary arenas for the unit’s quality improvement work, but others wanted even more arenas for sharing knowledge and experiences. Some participants also highlighted the co-location of different units (short-term, rehabilitation and municipal inpatient acute care units) as beneficial for intramunicipal collaboration and learning. These co-locations enabled collaborations between care staff, as they were able to make use of their competencies and functions. Such opportunities for collaboration and support from colleagues were significant during evenings or other times with low staffing. This exchange of knowledge and experience across units could strengthen the quality of the treatment provided to care recipients.

### Using Quality Indicators in Quality Improvement Work

Data from health registries (the Norwegian Registry for Primary Health Care and Municipality-State Reporting), and measurements from the national patient safety framework were examples of quality indicators and databases used in the services. All the study participants were in favour of using quality indicators. They all thought that such indicators were valuable instruments that could provide an overview of service quality. However, participants had varying knowledge of quality indicators as a concept and their databases. In addition, they used different indicators to varying degrees in the unit’s quality improvement work.

#### Reporting of Quality Indicators Varies

Various routines were implemented in the units to report quality measurements. Some units used quality indicators regularly, while others only measured when a change occurred in the recipient’s health status. Furthermore, the participants organised or facilitated meetings and seminars for development and training to ensure that the care staff knew why quality indicators should be used and how they should use them. Simultaneously, many participants highlighted their uncertainty about the Norwegian Registry for Primary Health Care database, as they had limited knowledge and time to use it. They also believed the database offered excessive opportunities for discretionary assessments, which could produce varying results when staff within the unit or other parts of the services screened the same care recipient. One participant emphasised the importance of knowing the recipients to be able to perform the screening:

*Sometimes we have brought in new registered nurses who suddenly run IPLOS* [the previous version of the Norwegian Registry for Primary Health Care] *on all the patients, but on what grounds? What have you done to examine the patient? ‘I just filled it in’, yes, but you have to base the screening on something and have a reason to do it.* (Participant 4)

Some participants explained that their units had a routine for copying previous screenings into a new electronic patient record note to enable documentation to be performed more quickly. However, a new screening was not always performed, and the results from the old screening were recorded in the new note.

#### Varying Use of Quality Indicators

Although all the participants endorsed using quality indicators, they had different perceptions of the quality indicators’ contribution towards the standardisation of service quality. Many participants believed that quality indicators were a key tool and essential to providing good and equitable services. In contrast, others did not use the indicators or were uncertain about their value. Participants emphasised results from quality indicator screenings, particularly those in the Norwegian Registry for Primary Health Care, as they could contribute to clarifying the status and progression of care recipients’ service needs. They used these screening results in meetings where services were allocated to care recipients, in conversations with next-of-kin, and when they prepared financial reports and/or accounts. Many participants emphasised that indicators had significant limitations regarding illuminating aspects of treatment and care while simultaneously highlighting some positive aspects. As one participant said,

. . . [the quality indicators] ensure equitable services. Similar thinking and similar understanding - that is a very positive thing. They should help to set a standard, then, for what we are supposed to do. I don’t think it’s a negative thing; it is just so much more for us than that single cross signifying that it’s ‘completed’. (Participant 1)

Other participants’ reflections supported this perspective, as they noted that quality indicators did not measure parts of the services that they thought were important. The care staff’s attitudes, ethics, values and good-naturedness were some factors the participants did not think were covered by quality indicators. Still, they were considered critical for guiding efforts to enhance service quality. In addition, differences in recipients’ care trajectories based on their diagnoses and treatment were an aspect that complicated the services’ opportunities to create indicators that suited all care recipients. Some participants proposed new quality indicators they believed could strengthen the importance of the indicator system. These new indicators were related to general aspects of the services, such as staffing standards and documentation, or specific clinical screenings, such as malnutrition and pressure ulcers, where the participants experienced the current indicators as deficient.

## Discussion

Our study shows that *delegation of responsibility*, *creation of comparable services* and *reporting and applying quality indicators* are central to how long-term care managers think about and incorporate quality improvement work in the context of service planning and provision. The first two themes can be considered crucial in developing and providing high-quality services. The third theme can be viewed as a feedback mechanism for evaluating how tasks and responsibilities are delegated, and consistent and comparable services are provided. The ‘Organising for Quality’ model developed by Bate et al. (2008) can be used to illuminate the long-term care managers’ approach to quality improvement work (Supplementary file 2). This model aims to uncover and identify factors and processes that can contribute to achieving and maintaining the quality of healthcare and has previously been utilised within the long-term care context (Johannessen et al., 2020). The model comprises six universal dimensions or challenges: *structural, political, cultural, educational, emotional*, as well as *physical and technological*, with different associated solutions to establish and maintain service quality (Bate et al., 2008). In addition, the model includes an *inner* and an *outer context*, each representing factors that can affect quality improvement work. Each of the study themes is discussed below in relation to this model and related research.

### Delegation of Quality Improvement Work in Complex Services

Long-term care managers’ delegation of quality improvement work to key nurses is confirmed by previous studies where key nurses and other staff were described as crucial for quality improvement work in long-term care (Asante et al., 2021; Ree et al., 2019). The quality improvement work delegated by managers can be demanding and complex, as it may include maintaining quality in the services’ day-to-day work and initiating new quality improvement initiatives. In light of Bate et al.’s (2008) model, delegating quality improvement work to key nurses may be a good strategy, as their competence and responsibilities can contribute to solving several challenges. One example of such a challenge is the implementation of quality systems to solve ‘structural challenges’ in quality improvement work. Using their competence, the key nurses can contribute to establishing well-functioning quality systems and guide other care staff in understanding and application of these systems. On the other hand, extensive delegation of quality improvement work from managers to key nurses may also be seen as a pulverisation of the managers’ responsibility for quality improvement work. It can reduce the managers’ opportunities to fulfil their statutory responsibilities for service quality (Ministry of Health and Care Services, 2017; The Norwegian Directorate of Health, 2017).

Key nurses’ balancing of responsibilities for quality improvement work on one side and organising service provision and delivery on the other was another essential finding. One important reason why the caseload of registered nurses in long-term care has grown in complexity in recent years is the transfer of responsibilities from specialist health service to municipalities, with resultant organisational restructuring and task shifting (Leong et al., 2021; St. Meld. 47, 2008–2009). Numerous studies indicate that long-term care staff are increasingly exposed to extensive workloads and time pressure (Glette et al., 2018; Melby et al., 2018; Yamamoto-Mitani et al., 2018). According to the managers in our study, the key nurses’ quality improvement work performance was challenged by their coexisting tasks in service management. However, their comprehensive responsibilities were also considered an advantage as it meant the nurses had an overview of the challenges in the services, which they could utilise in further quality improvement work initiatives. The balancing and administration of these complex tasks by key nurses can enhance service quality and maintain the unit’s administrative capacity (Ministry of Health and Care Services, 2017). The utilisation of the key nurses’ resources can also be linked to the structure and stability of the service, which is emphasised in the inner context of Bate et al.’s (2008) model. Consequently, with their competencies and function, the key nurses can contribute to creating continuity and improved systems for quality improvement work.

In Bate et al.’s (2008) model, staff is highlighted as one of the resources for improving the quality of the services. The staff are particularly emphasised in the solutions for handling ‘cultural’, ‘educational’ and ‘emotional’ challenges, for example, through emotional involvement in the unit’s quality improvement efforts. The managers in our study did not highlight care staff as crucial resources in the quality improvement work in a similar way. Still, they emphasised the care staff’s competence as decisive for the quality of direct care and treatment for the individual care recipient. This difference may indicate that the care staff in the units in our study may have been a somewhat underutilised resource in long-term care service planning and provision. Thus, there is a potential for managers to use these staff members to a greater extent in the unit’s quality improvement work.

The decentralisation of health service tasks from specialist services to municipalities and the expanding number of care recipient groups with complex health needs have also increased the demand for and importance of long-term care staff skills and competence (St. Meld. 47, 2008–2009). Previous studies have found that staff competence is decisive in developing and maintaining high-quality long-term care (Asante et al., 2021; Johannessen et al., 2020) and that staff with specialist knowledge or expertise act as ‘resource persons’ for defined areas of responsibility such as hygiene, nutrition and palliative care. In our study, defined care staff with special responsibilities for the units’ quality improvement work was mentioned only to a minimal extent. Additionally, the managers did not express any desire to increase the involvement of care staff in this work. However, including and delegating responsibility for parts of quality improvement work to (groups of) care staff may be a measure that can promote competence development and engagement among employees. In addition, this approach could lead to better distribution of tasks between the care staff and key nurses. Increasing the division of tasks between regular staff and key nurses may, therefore, be a measure that managers can use in the units’ quality improvement work.

### Use of Quality Systems in Continuously Changing Services

Standardisation of care and treatment, through comparisons across units and municipalities, as well as development of procedures and collaboration were approaches the managers in our study used to improve service quality. Such approaches align with national and international ambitions (Institute of Medicine, 2001; The Norwegian Directorate of Health, 2017) and are suggested as solutions to ‘cultural’, ‘structural’ and ‘educational’ challenges in the model by Bate et al. (2008). While managers emphasised the importance of comparable services, they also expressed that the quality systems provided limited knowledge on care quality. High workloads among managers, various reorganisations, and task transfers may explain their lack of knowledge, time and opportunities to obtain more information from the system. In addition, the quality system in Norwegian healthcare is under development, and such implementation requires time and effort from the entire organisation (Johannessen et al., 2020; Tikkanen et al., 2020). Therefore, managers’ overview of such measures is likely to improve over time. A better overview can also give managers a basis for providing input on any additional measures that they believe can strengthen the basis for comparison across and within municipalities.

Other approaches to standardisation, such as procedures for reaching quality aims, were also highlighted by the managers in our study. However, the units used these procedures infrequently due to insufficient time and resources. Similar challenges to implementing and applying measures aimed at standardising have been reported nationally and internationally (Alexander et al., 2022; Johannessen et al., 2020). At the same time, quality procedures can be viewed as solutions to ‘structural’ challenges (Bate et al., 2008). Previous research has shown that managers in long-term care services view quality procedures as a way of ensuring high-quality services (Asante et al., 2021). According to this study, procedures must be flexible and easy to understand to be functional for the unit’s care staff. Although our findings indicate that units used such procedures only to a small extent, they may be valuable in future quality improvement work.

### Valuation and Uncertainty of Quality Indicators

The focus on quality indicators in healthcare services is increasing (OECD, 2019; WHO & UN, 2022). Measurements, indicators and benchmarks are also highlighted by Bate et al. (2008) as a part of evidence-based learning and as solutions to ‘educational’ challenges. Our findings indicate that long-term care managers considered quality indicators valuable but that current indicators did not measure essential parts of recipients’ care and treatment. The managers also reflected on whether all aspects of the services could be measured or whether some aspects were ‘unmeasurable’. This finding is consistent with the conclusions of previous research on quality indicators, where quality has been described as more than concrete or quantifiable (Aase et al., 2021; Tøssebro et al., 2022). In Norway, the use of quality indicators is part of the statutory obligations of healthcare service managers to provide professionally sound services (Saunes et al., 2020; The Norwegian Directorate of Health, 2017). Using quality indicators as a strategy for evaluating and determining service quality is, therefore, also a response to regulations imposed by the legislature. Thus, managers must continue to acquire knowledge about quality indicators and their application in clinical practice. However, the alignment of legislative requirements with clinical needs, as emphasised by the managers in this study, may be an approach towards measuring the areas of importance to quality improvement in a clinical setting.

How managers organised the reporting of the indicators and trained care staff in screening and reporting varied considerably across the units included in our study. There may be several reasons for these variations. Previous research in the long-term care context has shown that a lack of time and resources and a high number of part-time care staff may explain the shortfall of staff training, patient screening and care documentation (Johannessen et al., 2020; Ree et al., 2019). Shift work and part-time care staff can make it challenging for managers to conduct training, including quality indicator screening and reporting. In addition, part-time care staff may not have the same opportunities as full-time care staff to perform as many quality indicator screenings and reporting. Challenges related to competence development in long-term care are common, as documented in a previous study (Soreide et al., 2019). According to Bate et al.’s (2008) model, challenges related to competence development are identified as ‘political’ and ‘educational’ challenges, and external partners and knowledge acquisition are two proposed solutions. Such dialogues and exchanges of experience between long-term care managers and other managers in the services may strengthen the organisation of quality indicator reporting further. Furthermore, such dialogues can contribute to creating common approaches and routines for care staff training and the implementation of quality indicator reporting, which can be useful in long-term service quality improvement work.

### Strengths and Limitations of This Study

This study has several strengths. Managers working at various levels of long-term care services participated in the study, which enabled a breadth of experiences with quality improvement work to be included. Furthermore, data were collected through in-depth interviews. This method allowed us to explore the managers’ multifaceted quality improvement work in detail, which provided us with rich material. During the interviews, the managers described their approaches to and perspectives on quality improvement work and how they worked to strengthen the quality of care in services. However, it is possible that what they said did not accurately reflect all aspects of the services’ quality improvement work. This possibility could be a limitation of the study. Using participant observation, in addition to the interviews, could have enabled us to uncover such variations and strengthen the trustworthiness of the findings. The recruitment process may also be a limitation of the study since we do not have any information about non-participants.

## Conclusions

Our study indicates that managers delegate several tasks and responsibilities for quality improvement work to key nurses and view their roles as crucial. Comparing services, collaborating within and across units and municipalities, and applying quality indicators are also essential approaches to long-term care managers’ quality improvement work. Nevertheless, these measures seem to be implemented to varying degrees due to a lack of time, care staff and service reorganisations. An improved and optimised delegation of quality improvement work tasks could be a valuable approach for long-term care managers. It could strengthen the managers’ opportunities to take care of their quality improvement work obligations, facilitate and assign more tasks to the care staff and identify and prioritise appropriate responsibilities for key nurses. Cooperation and exchanges of experience among managers within and among municipalities could also be an approach managers can use to strengthen the service’s quality improvement work. Further research should explore other aspects of long-term care managers’ facilitation of high-quality care. One possible avenue for future research could be exploring in more depth how managers conceptualise their responsibilities and perceive their role in leading quality improvement work within the services.

### Implications for Nursing Managers

The findings of this study provide insights into long-term care managers’ approaches to creating high-quality services. Our findings highlight that the competencies and resources of the unit’s care staff could be used to a greater extent in the unit’s quality improvement work. Better integration of quality improvement work into the care staff’s day-to-day work could reduce the workload faced by key nurses and strengthen the focus on quality improvement work through the unit’s entire staff group. By using collaboration and knowledge exchange as a part of quality improvement work, managers could learn from each other and exchange examples of best practices. These collaborations could also allow managers to acquire more knowledge about quality indicator systems, apply indicator results more effectively, and propose system improvements to authorities.

## Supplemental Material

sj-docx-1-gqn-10.1177_23333936251336093 – Supplemental material for Long-Term Care Managers’ Approaches to Quality Improvement Work in Service Planning and Provision: A Qualitative Interview Study Supplemental material, sj-docx-1-gqn-10.1177_23333936251336093 for Long-Term Care Managers’ Approaches to Quality Improvement Work in Service Planning and Provision: A Qualitative Interview Study by Randi Olsson Haave, Marianne Sundlisæter Skinner and Line Melby in Global Qualitative Nursing Research

sj-pdf-2-gqn-10.1177_23333936251336093 – Supplemental material for Long-Term Care Managers’ Approaches to Quality Improvement Work in Service Planning and Provision: A Qualitative Interview Study Supplemental material, sj-pdf-2-gqn-10.1177_23333936251336093 for Long-Term Care Managers’ Approaches to Quality Improvement Work in Service Planning and Provision: A Qualitative Interview Study by Randi Olsson Haave, Marianne Sundlisæter Skinner and Line Melby in Global Qualitative Nursing Research
