# Supplementary figures and images for: Long-Term Care Managers’ Approaches to Quality Improvement Work in Service Planning and Provision: A Qualitative Interview Study
Source: Glob Qual Nurs Res. 2025 Apr 28;12:23333936251336093. doi: 10.1177/23333936251336093 (PMC12038208; doi:10.1177/23333936251336093)

**
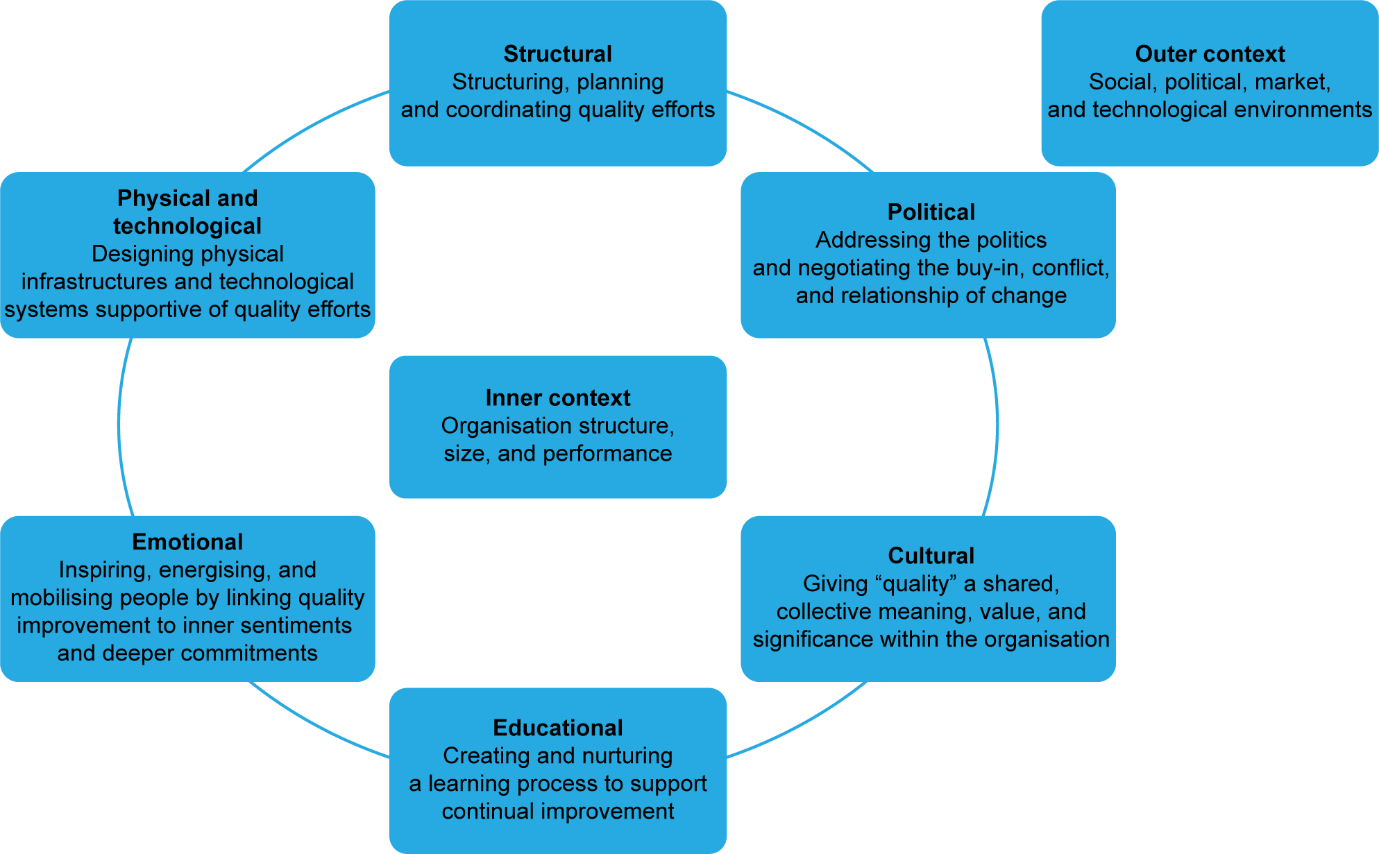
**

**Figure 1.** Organising for Quality (based on the framework by Bate, Mendel and Robert 2008)

Supplement: sj-docx-1-gqn-10.1177_23333936251336093 – Supplemental material for Long-Term Care Managers’ Approaches to Quality Improvement Work in Service Planning and Provision: A Qualitative Interview Study [file sj-docx-1-gqn-10.1177_23333936251336093.docx]
