# Supplementary material for: Long-Term Care Managers’ Approaches to Quality Improvement Work in Service Planning and Provision: A Qualitative Interview Study
Source: Glob Qual Nurs Res. 2025 Apr 28;12:23333936251336093. doi: 10.1177/23333936251336093 (PMC12038208; doi:10.1177/23333936251336093)
Supplement: sj-pdf-2-gqn-10.1177_23333936251336093 – Supplemental material for Long-Term Care Managers’ Approaches to Quality Improvement Work in Service Planning and Provision: A Qualitative Interview Study [file sj-pdf-2-gqn-10.1177_23333936251336093.pdf]

Interview guide

Introduction

The informants introduce themselves and their position. How long have you worked in this position and at this unit? What is your area of responsibility in this position?

Startup point 1, Units quality work.

Can you give me examples of the focus of the unit's quality work/quality improvement work? Can you tell me about the background for this focus(es)? What are your focus and responsibilities in the quality improvement work? Do you or the unit collaborate with other units or partners in this work?

Startup point 2, Quality and quality indicator as concepts.

What does quality as a term mean for you? What does the term quality indicator mean for you? How are these indicators used in the unit's daily work? Can you tell me how you experience the quality indicators? Do you feel that the quality indicators and the quality improvement work describe the care staff's work? How do you think the quality indicators affect the care provided to the recipients? What are the benefits of the quality indicators and the quality improvement work? Does it have any disadvantages?

Startup point 3, Care recipients

Can you tell me about the unit routines when you receive a new care recipient? Can you give me examples of social activities provided for the care recipients? How do you work with nutrition and prevention of malnutrition at the unit? Do the care staff use nutrition screening tools? How are meals arranged and served in the units? Does the unit involve the care recipient in the menu and the meal context?

Startup point 4, User participation

Does the unit use any surveys or other kinds of user involvement? Can you tell me about the preparation and application of the user surveys?
